# Modeling the spreading and interaction between wild and transgenic mosquitoes with a random dispersal

**DOI:** 10.1371/journal.pone.0205879

**Published:** 2018-10-31

**Authors:** Ana Paula Wyse, Antonio José Boness dos Santos, Juarez dos Santos Azevedo, Josenildo Silva de Lima, Jairo Rocha de Faria

**Affiliations:** 1 Department of Scientific Computing, Informatic Center, Universidade Federal da Paraíba, João Pessoa, Paraíba, Brazil; 2 Exact and Technology Sciences Center, Universidade Federal do Recôncavo of Bahia, Cruz das Almas, Bahia, Brazil; 3 Graduate Program in Mathematical and Computational Modeling, Informatic Center, Universidade Federal da Paraíba, João Pessoa, Paraíba, Brazil; Shandong University of Science and Technology, CHINA

## Abstract

Due to recent advances in genetic manipulation, transgenic mosquitoes may be a viable alternative to reduce some diseases. Feasibility conditions are obtained by simulating and analyzing mathematical models that describe the behavior of wild and transgenic populations living in the same geographic area. In this paper, we present a reaction–diffusion model in which the reaction term is a nonlinear function that describes the interaction between wild and transgenic mosquitoes, considering the zygosity, and the diffusive term that represents a nonuniform spatial spreading characterized by a random diffusion parameter. The resulting nonlinear system of partial differential equations is numerically solved using the sequential operator splitting technique, combining the finite element method and Runge-Kutta method. This scheme is numerically implemented considering uncertainty in the diffusion parameters of the model. Several scenarios simulating spatial release strategies of transgenic mosquitoes are analyzed, demonstrating an intrinsic association between the transgene frequency in the total population and the strategy adopted.

## Introduction

Vector-borne diseases have always been of great concern to populations and government authorities in countries with a tropical climate, especially those with a low human development. The combination of favorable climate and precarious sanitation provides ideal environmental conditions for the proliferation of many vectors causing various types of diseases that affect the populations of these regions, leading to considerable economic and social disruption. Due to climate change, intensive farming, dams, irrigation, deforestation, population movements, rapid unplanned urbanization, and increases in international travel and trade [1], the incidence of tropical diseases has been extended to regions beyond the tropics, making such diseases a worldwide focus.

Malaria is the best-known and most deadly of the vector-borne diseases. According to the World Health Organization [2], in 2016, 91 countries reported a total of 216 million cases of malaria, resulting in 445,000 deaths, approximately the same number as was reported in 2015. However, among the vector-borne diseases, the incidence of dengue has the fastest growth in the world, with a 30-fold increase over the past 50 years. In addition to malaria and dengue, yellow fever, chikungunya, zika, and others are also prominent.

Vector control is the primary method to prevent and reduce the transmission of these diseases. Combined strategies are strongly recommended to achieve the greatest benefit of controlling the proliferation of diseases, minimizing impacts to the ecosystem as well as adverse effects on public health. Therefore, it becomes necessary to understand the local vector ecology and the local patterns of disease transmission so that we can choose the appropriate vector control tools from the wide range of available options.

With recent advances in the genetic manipulation of mosquitoes, in particular, *Aedes spp*. and *Anopheles spp*., there has been considerable enthusiasm in the search for novel vector control approaches to prevent a wide variety of diseases. In this way, the use of genetically modified mosquitoes can be a promising alternative to control mosquito–borne infections in addition to the traditional prophylaxis, vaccines, medication, and insecticides.

The conception of disease control by genetic manipulation is not new; the concept arose in the 1930s-40s for the control of arthropod vectors by three independent research pioneers: A. S. Serebrovskii [3], F. L. Vanderplank [4] and E. F. Knipling [5]. Considerable research on genetic strategies to control mosquitoes was conducted during the 1970s (e.g., see Curtis et.al [6]) on mosquito populations with sterile males. A review of the applications of genetics to vector control can be seen in Refs. [7–9].

The notion of increasing a gene frequency in a vector population so that it interferes with a pathogen has made substantial progress, e.g., Collins and James [10], whose hypothesis resulted in the reduction or elimination of the transmission of that pathogen.

Laboratory-based discovery research is now underway on a variety of genetic engineering strategies for controlling arthropod vectors of disease, producing mosquitoes that, when released, reproduce with wild mosquitoes and cause their offspring to be refractory to diseases [11, 12], unable to detect CO_2_ [13], carrying a lethal gene [14], sterile [9] or infected by *Wolbachia pipientis*, an endosymbiotic bacteria able to inhibit viral replication in the mosquito [15] and to reduce its lifespan [16]. Genetically modified mosquitoes refractory to malaria, for example, were first obtained in 2002 using a technique developed by Catteruccia et al. [17]. To obtain them, scientists have developed two different types of transgenic mosquitoes of species *Anopheles stephensi* using the CP (carboxypeptidase) promoter: one of them expresses the synthetic peptide SM1 (salivary gland and midgut binding peptide 1) [11], while the other expresses the enzyme PLA2 (phospholipase A2) present in bee venom [12]. In 2010, Amenya et al. [18] used this technique to obtain *A*. *stephensi* transgenic lines containing *ϕ*C31 attP “docking” sites linked to a fluorescent marker gene. More recently, in 2015, Gants and Bier [19] developed a method based on the self-propagating CRISPR/Cas9 genome editing technology, which converts heterozygous to homozygous mutations. This artificial drive mechanism, called a mutagenic chain reaction (MCR), was tested for *Drosophila melanogaster* with a 97% efficiency. Next, MCR inheritance proved to be efficient to *A*. *stephensi*, although lines obtained by mating between drive-males and wild-type females presented a higher drive efficiency than lines obtained by mating between drive-females and wild-type males [20, 21].

Assuming these ideas and linking them with mathematical models, the dynamics governing the interaction between wild and transgenic mosquitoes has been subsequently studied. For example, in 2004, Li presented a discrete system model that considered the interaction between two varieties of mosquitoes: wild and transgenic, without the distinction of zygosity [22]; a continuous version was obtained in 2007 [23], and a model considering the impact of transgenic mosquitoes on malaria transmission was presented in 2011 [24]. In addition, a discrete time model, without the distinction of zygosity and taking into account only the horizontal and vertical transmission of a genetically modified bacterium was proposed by Li [25]; in this case, the horizontal transmission depends on mating between wild mosquitoes and those that have mutated due to contact with the bacteria. Continuous modeling with the distinction of zygosity was proposed by Wyse et al. [26, 27], based on the initial sketch for the interaction between three varieties of mosquitoes: wild, heterozygous and homozygous transgenic [28]. A nondimensional version was considered in 2011, in which the fitness of wild and transgenic mosquitoes was assumed to be distinct [29].

In 2015, Unckless et al. [30] adopted a Wright-Fisher model, with random mating in a population of infinite size, to determine the existence and stability of an internal equilibrium when there is a fitness cost to the MCR allele [19], resulting in conditions for successful gene fixation and invasion. In 2018, Noble et al. [21] proposed a stochastic, Moran-based model for evaluating the dissemination efficiency of the self-propagating homing drives in finite wild populations considering the resistant alleles, suggesting its use to suppress wild populations of mosquitoes transmitting malaria.

Strategies for delivering malaria-resistant [31, 32] and sterile genetically modified mosquitoes [33] used techniques of optimal and inundative control, respectively. Studies such as these allow for the achievement of sustainable genetic control programs, help public health agencies in making decisions, and indicate the amount of transgenics required, which genetic composition is most appropriate, and the ideal and local timing for release, as well as cost implications.

In this paper, we propose a new mathematical model that describes the dynamics of the interaction by the mating between transgenic and wild-type mosquitoes, as well as the spreading of the transgene that determines the interruption of an epidemiological process. For this purpose, the transgenic mosquitoes are differentiated according to their zigozity, being heterozygous or homozygous. The interaction between mosquitoes describes a density dependence effect for vital rates and imposes a maximum limit of population growth that occurs according to the carrying capacity. In addition, all populations are in absolute numbers, preserving parameters that can describe effects such as seasonality, stochasticity, etc. The spreading is governed by Fick’s law, with a random diffusion coefficient. Thus, the mathematical model obtained is a nonlinear reaction-diffusion system with uniformly distributed parameters. This resulting system is solved by operator splitting methods that are well known in problem solving that result in large systems of partial differential equations, as well as problems involving nonlinear chemical reactions [34], mosquito dispersal [35], nonlinear Schrodinger applications [36] and others. Sequential and iterative splitting for nonlinear problems with locally Lipschitz-continuous operators, which generates the lowest splitting error and convergence of first order, was presented and verified in [37]. We use the sequential operator to split the system into two parts and solve each one separately with specialized numerical techniques, in this case, combining the well-known fourth order Runge-Kutta method for the nonlinear dynamic problem and the space-time finite element method with distributed parameters for the diffusion problem. The numerical solution obtained, from different initial conditions, show some of the possible scenarios to be discussed to investigate the spreading of transgenic mosquitoes, their behavior with uncertainty parameters and guidelines for future investigations.

## Materials and methods

This section describes population dynamics resulting from the introduction of genetically modified mosquitoes into wild-type populations. The proposed mathematical model is based on strategies aimed at the genetically modified mosquitoes that are engineered to have a reduced transmission capacity of a particular pathogen and are fertile and able to propagate and perpetuate their inheritable trait in the wild mosquito population. For this, we consider transgenic mosquitoes of the genus *Anopheles*, more precisely *A*. *stephensi*. Studies have shown a reduction in the fitness of this mosquito in relation to the wild one when homozygous transgenic lines were used [38] and no significant effect when the heterozygous lines were used [18, 39, 40]. One of the known factors that can result in reduced fitness is inbreeding depression associated with the rearing of transgenic mosquitoes as homozygotes. *A*. *stephensi* is the key vector of malaria in urban areas of India, a country responsible for six percent of the world’s malaria cases in 2016 (216 million confirmed cases), according to the World Malaria Report 2017 [2]. In many Indian towns, this mosquito has becomes truly domestic [41].

There is increasing evidence of the adaptation of anophelines to organically polluted water bodies [42]. Studies show preferences to deep water as a breeding habitat [43] but also a diversity of habitats, with activity in fresh or salt water marshes, mangrove swamps, rice fields, grassy ditches, the edges of streams and rivers and small, temporary rain pools, which can be the result of human behaviors such as broken water pipes, cans, poorly maintained drains, overhead tanks, truck tire tracks on unpaved roads, and low lying areas that are liable to flooding among others [44–46]. Based on this information, the mathematical model will assume the simplified hypothesis that females are able to find a breeding in any place of the domain considered, obtaining equal reproductive success. This makes sense in urban areas, for example, where the incidence of malaria is evident [41].

The dispersal distance of mosquitoes in urban areas is considerably less than in open environments, with the local topography being one of the several extrinsic and intrinsic factors influencing the dispersal of mosquitos. Different values for the dispersal of mosquitoes can be found in the literature, varying due to meteorological conditions, topography, physiological status of released mosquitoes, body size, population density, availability of oviposition and resting sites. Studies conducted by Bailey et al. [47] concluded that the short distance dispersal of *Culex tarsalis* was independent of wind direction, whereas longer distance dispersal (> 5 km) was always downwind. This finding gives us guidelines of the possibility of this fact occurring for other mosquito species (Diptera: *Culicidae*). Being that the premise of the proposed model is based on urban mosquito dispersal, no advection will be considered.

### Mathematical modeling of the mosquito dispersal

Let *u*_*i*_, *i* = 1, 2, 3, denote populations of wild, heterozygous and homozygous transgenic mosquitoes. If we assume that the movement of individual mosquitoes is similar to Brownian motion in a nonuniform field, then we can define the rate of change of the mosquito density at time *t* ∈ *Ī*_*t*_ ≡ (0, T], *T* > 0, as

$$\begin{array}{c} \frac{\partial u_{i}\left(x,t\right)}{\partial t}=\kappa_{i}\frac{\partial^{2}u_{i}\left(x,t\right)}{\partial x^{2}}\;x\in {\bar{I}}_{d}, \end{array}$$ (1)

where ${\bar{I}}_{d}=\left[0,L\right]\subset I\;\;I\;R$ is the spatial domain, with *L* > 0, *κ*_*i*_ denotes the random diffusion rate coefficients of both wild and transgenic mosquitoes. These coefficients aim to evolve a probabilistic response, creating a random heterogeneous medium. We also assume that the boundary of region *Ī*_*d*_ does not permit mosquito transport, and thus, we have zero flux boundary conditions

$$\begin{array}{c} \frac{\partial u_{i}}{\partial x}\left(0,t\right)=\frac{\partial u_{i}}{\partial x}\left(L,t\right)=0. \end{array}$$ (2)

This environment affecting mosquitoes’ dispersal behavior was considered by Lutambi et al. [48], and the change in mosquito density was described by a discrete-space continuous-time mathematical model. Additionally, Dufourda and Dumont [35] showed that environmental parameters, such as vegetation, may have an intrinsic influence on mosquito distribution and the efficiency of vector control tools. Taking into account the relationship between environmental variability and the dispersal of mosquitoes, it is feasible to consider diffusion coefficients that are not deterministic parameters.

The mathematical model assumes that populations *u*_*i*_ have the same fitness, according to studies about *A*. *stephensi* [39], ensuring equal reproductive success and reflecting how well transgenic mosquitoes are adapted to the wild environment, with the ability to compete and mate. Each individual progresses and emerges into adulthood at a rate *ϵ* and leaves due to mortality. Assuming a stable environment, competition for resources may occur in immature forms, implying losses in adulthood [49] and leading to density-dependent mortality $\frac{\gamma \sum u_{i}}{C}$, with *γ* being the intrinsic rate of growth, *C* the carrying capacity, and ∑*u*_*i*_ = *u*_1_ + *u*_2_ + *u*_3_, or natural death *δ*. These processes account for the dynamics of each subgroup over time. Since allele combinations are equally likely to occur, the matings are random and a Punnett Square can be a tool to predict the probability of a mating producing each genotype [50]. Because mosquitoes are diploid organisms, it is proper to establish a wild mosquito’s genotype as (*w*, *w*) and homozygous transgenic genotype as (*g*, *g*). After crossing, we established homozygous populations (*w*, *w*) and (*g*, *g*) and a heterozygous population (*w*, *g*), as seen in Table 1. The dominance of *w* or *g* defines the phenotype of the heterozygous mosquitoes and depends on genetic manipulation, but it is outside the scope of this paper.

**Table 1:** Genotypic offspring frequencies obtained from the mating between wild, heterozygous and homozygous transgenic mosquitoes.

Genotype	Mating					
	*u*_1_ × *u*_1_	*u*_1_ × *u*_2_	*u*_1_ × *u*_3_	*u*_2_ × *u*_2_	*u*_2_ × *u*_3_	*u*_3_ × *u*_3_
(*w*, *w*)	*a*_11_	*a*_12_	*a*_13_	*a*_22_	*a*_23_	*a*_33_
(*w*, *g*)	*b*_11_	*b*_12_	*b*_13_	*b*_22_	*b*_23_	*b*_33_
(*g*, *g*)	*c*_11_	*c*_12_	*c*_13_	*c*_22_	*c*_23_	*c*_33_

Let *a*_*ij*_, *b*_*ij*_ and *c*_*ij*_ denote genotypic frequencies for *u*_1_, *u*_2_ and *u*_3_ obtained from mating *u*_*i*_ × *u*_*j*_, *i*, *j* = 1, 2, 3. These coefficients must satisfy the relation *a*_*ij*_ + *b*_*ij*_ + *c*_*ij*_ = 1.

From the above assumptions, the dynamics of interactions between wild and transgenic mosquitoes with spreading can be established by the following spatiotemporal model with uncertainty parameters:

Let (Ω; F; *P*) be a probability space in which the variations of the random inputs exhibit, where Ω is the set of outcomes, $F\subset 2^{\Omega }$ is the *σ*-algebra of events and P:F→[0,1] is a probability measure. Given $\{a_{ij},b_{ij},c_{ij},\epsilon ,\gamma ,C,\delta \}\in I\;R$, the model problem is to find the population of mosquitoes $u_{i}(x,t,w)\in I\;R$ for all *x* ∈ *Ī*_*d*_, *t* ∈ *Ī*_*t*_ and *ω* ∈ Ω that satisfy the following system of nonlinear boundary value problem with random diffusion coefficients

$$\begin{array}{c} \left\{ \begin{array}{cc} \frac{\partial u_{1}}{\partial t} & =\kappa_{1}\left(\omega \right)\frac{\partial^{2}u_{1}}{\partial x^{2}}+(\frac{\epsilon }{\sum u_{i}}-\frac{\gamma }{C})\sum \sum a_{ij}u_{i}u_{j}-\delta u_{1},\\ \frac{\partial u_{2}}{\partial t} & =\kappa_{2}\left(\omega \right)\frac{\partial^{2}u_{2}}{\partial x^{2}}+(\frac{\epsilon }{\sum u_{i}}-\frac{\gamma }{C})\sum \sum b_{ij}u_{i}u_{j}-\delta u_{2},\\ \frac{\partial u_{3}}{\partial t} & =\kappa_{3}\left(\omega \right)\frac{\partial^{2}u_{3}}{\partial x^{2}}+(\frac{\epsilon }{\sum u_{i}}-\frac{\gamma }{C})\sum \sum c_{ij}u_{i}u_{j}-\delta u_{3}, \end{array}\right. \end{array}$$ (3)

with Neumann boundary conditions given by

$$\begin{array}{c} \frac{\partial u_{i}}{\partial x}\left(0,t\right)=\frac{\partial u_{i}}{\partial x}\left(L,t\right)=0, \end{array}$$ (4)

and initial conditions

$$\begin{array}{c} u_{i}\left(x,0\right)={\bar{u}}_{i}\left(x\right), \end{array}$$ (5)

Here the diffusion coefficients *κ*_*i*_ are uniformly distributed random fields in the interval $[s_{i},s_{f}]\subset I\;R$, written as *κ*_*i*_ ∼ *U*(*s*_*i*_, *s*_*f*_). These coefficients are used to improve the computational model and to calibrate a known parameter in order to build a regularized solution *u*_*i*_.

Disregarding the diffusion and adding up the three eq (3), the total mosquito population is described by the Verhulst–Pearl logistic equation with harvesting

$$\begin{array}{c} \frac{dN}{dt}=\gamma N(1-\frac{N}{C})-\delta_{2}N, \end{array}$$ (6)

where *N* = ∑*u*_*i*_ is the total population of mosquitoes and *γ* = *ϵ* − *δ*_1_, where *ϵ* is the rate of the emergence of mosquitoes into adulthood, and *δ*_1_ is the mortality rate due to natural causes. The harvesting effect represented by the density-independent mortality rate *δ*_2_ ensures the stabilization of the population below the carrying capacity *C*, more precisely, at the nonnull equilibrium point

$$\begin{array}{c} N*=C(1-\frac{\delta_{2}}{\gamma }). \end{array}$$ (7)

The mosquitoes’ population dynamics described by Eq (6) was fitted to field data for some regions [28, 51] with accurate results. Mathematical models considering variable mosquito populations were used in [52, 53] and [51], suggesting the logistic form to an intraspecific competition. Considering diffusion in Eq (6), we obtain the Fisher-Kolmogorov model with harvesting.

For results that guarantee the model governed by system (3) without dispersal is biologically and mathematically well-posed, see S1 Appendix.

### Numerical Formulation

In this section, we focus on the development of discrete formulations and applications of computational techniques to numerically solve the proposed model. For this purpose, we present an operator splitting method [54] used to solve the nonlinear reaction-diffusion system (3). The idea is to decouple the original system into another equivalent system, generating a combination of two distinct physical subsystems formed by problems of less complexity. The advantage of adopting this type of strategy is that we can prevent systems that require costly operations and have the possibility to choose the most optimized algorithms for each operator.

Let us consider the sequential operator-splitting method, which solves two subproblems sequentially on each time interval and connects them via the initial conditions. To describe the developed algorithm, we proceed with a natural decomposition of the system (3) into two coupled new problems: a system of partial differential equations with the purely diffusive term and a system of nonlinear differential equations associated with the purely reactive term. To solve the diffusive problem, we use the semidiscrete finite element method with an implicit finite difference scheme of the Crank-Nicolson type. The second system, which is nonlinear, is solved using the fourth-order Runge-Kutta method for its high accuracy. The uncertainty on the diffusion parameter is quantified using the Monte Carlo method.

Introducing the temporal discretization ${\bar{I}}_{t}=\left[0,T\right]={⋃}_{n=0}^{N}I_{t_{n}}$, such that $I_{t_{n}}=\left[t_{n},t_{n+1}\right]$ is a partition of *I*_*t*_, and *N* = *T*/Δ*t* is the number of partitions of *I*_*t*_ with an uniform time step Δ*t* = *t*_*n*+1_ − *t*_*n*_, we follow the procedure below:

***Step 1***: For the initial time, *t* = *t*_0_, initialize the variables *u*_*i*_(*x*, *t*_0_) = *ū*_*i*_(*x*) for each *i* = 1, 2, 3.
***Step 2***: For some fixed *n* ≥ 0, considering known the initial conditions *u*_*i*_(*x*, *t*_*n*_) and defining *ŭ*_*i*_(*t*_*n*_) = *u*_*i*_(*x*, *t*_*n*_), we calculate *ũ*_*i*_(*x*, *t*) in the time step *t*_*n*+1_ through the following problem:
  **Problem A**: Given *κ*_*i*_: Ω → (0, ∞), to find ${\tilde{u}}_{i}\left(x,t,\omega \right)\in I\;R$, with *x* ∈ *Ī*_*d*_, $t\in I_{t_{n}}$ and *ω* ∈ Ω, satisfying the following system:
    $$\begin{array}{c} \frac{\partial {\tilde{u}}_{i}\left(x,t,\omega \right)}{\partial t}=\kappa_{i}\left(\omega \right)\frac{\partial^{2}{\tilde{u}}_{i}\left(x,t,\omega \right)}{{\partial x}^{2}}, \end{array}$$ (8)
    with boundary conditions
    $$\begin{array}{c} {\left({\tilde{u}}_{i}\right)}_{x}\left(0,t\right)={\left({\tilde{u}}_{i}\right)}_{x}\left(L,t\right)=0, \end{array}$$ (9)
    and initial conditions
    $$\begin{array}{c} {\tilde{u}}_{i}\left(x,t_{n}\right)={\overset{˘}{u}}_{i}\left(t_{n}\right). \end{array}$$ (10)
***Step 3***: In the same time interval $I_{t_{n}}$, we use the solution of Problem A, given by the previous step, as the initial condition to calculate the solution of the system of coupled nonlinear ordinary differential equations, associated with the model (3), expressed by the following problem:
  **Problem B**: Given $\{a_{ij},b_{ij},c_{ij},\gamma ,C,\varepsilon ,\delta \}\in I\;R$, find $u_{i}(t)\in I\;R$, $t\in I_{t_{n}}$ satisfying the following system:
    $$\begin{array}{c} \left\{ \begin{array}{cc} \frac{du_{1}}{dt} & =(\frac{\epsilon }{\sum u_{i}}-\frac{\gamma }{C})\sum \sum a_{ij}u_{i}u_{j}-\delta u_{1},\\ \frac{du_{2}}{dt} & =(\frac{\epsilon }{\sum u_{i}}-\frac{\gamma }{C})\sum \sum b_{ij}u_{i}u_{j}-\delta u_{2}\\ \frac{du_{3}}{dt} & =(\frac{\epsilon }{\sum u_{i}}-\frac{\gamma }{C})\sum \sum c_{ij}u_{i}u_{j}-\delta u_{3}, \end{array}\right. \end{array}$$ (11)
    with initial condition
    $$\begin{array}{c} u_{i}\left(t_{n}\right)={\tilde{u}}_{i}\left(x,t_{n+1}\right), \end{array}$$ (12)
    where *ũ*_*i*_(*x*, *t*_*n*+1_) are the solutions obtained from Problem A.
***Step 4***: The solution of Problem B is given by the approximate solution of the model at $t_{n+1}\in I_{t_{n}}\subset I_{t}$. If *t*_*n*+1_ < *T*, we increment *n*, return to step 2, and repeat the process until equality occurs.

To solve Problem A, we adopted a finite element method with uniformly distributed parameters using a Monte Carlo scheme to obtain the first and second moment of the solution. The spatial domain is discretized using an uniform partition *I*_*e*_ with *n*_*e*_ elements, such that ${\bar{I}}_{d}={⋃}_{e=1}^{n_{e}}I_{e}$ and ${⋂}_{e=1}^{n_{e}}I_{e}=\emptyset$. The method seeks an approximation ${\tilde{u}}_{i}^{h}\left(x,t,\xi^{\left(k\right)}\right)$ to ${\tilde{u}}_{i}\left(x,t,\omega \right)$ in the Lagrangian finite element space of class *C*^0^(*I*_*d*_) and degree *l* given by $V_{h}=\left\{v^{h}\left(x,t\right)\in C^{0}\left(I_{d}\right)|v^{h}\left(0\right)=v^{h}\left(L\right)=0,\forall \;v^{h}\left(I_{e}\right)\in {\hat{P}}_{l}\left(I_{e}\right)\right\}$.

It is defined by requiring that, for each realization on the diffusion parameter {*κ*_*i*_(*ξ*^(*k*)^) ∈ [*κ*_*min*_, *κ*_*max*_]} (*k* = 1, …, *N*_*s*_) and for all *v*^*h*^ ∈ *V*_*h*_,

$$\begin{array}{c} \int_{I_{d}}\frac{\partial {\tilde{u}}_{i}^{h}v^{h}}{\partial t}dx+\int_{I_{d}}\kappa_{i}\left(\xi^{\left(k\right)}\right)\frac{\partial {\tilde{u}}_{i}^{h}}{\partial x}\frac{\partial v^{h}}{\partial x}dx=0. \end{array}$$ (13)

Concerning Problem B given by the ODE system Eq (11), we use the classical fourth-order Runge-Kutta method to obtain its solution.

Finally, the statistical moments of the discrete variables are computed by generating a population of *N*_*s*_ samples $\left\{{\tilde{u}}_{i}^{n}\left(\cdot ,\xi^{\left(k\right)}\right),\;k=1,\ldots ,N_{s};i=1,2,3\right\}$ corresponding to the problems *A* and *B*. The mean and variance are computed using the approximations

$$\mu ({\tilde{u}}_{i})=\frac{1}{N_{s}}\overset{N_{s}}{\underset{k=1}{\sum }}{\tilde{u}}_{i}\left(\cdot ,\xi^{\left(k\right)}\right)$$ (14)

$$\sigma^{2}({\tilde{u}}_{i})=\frac{1}{N_{s}}\overset{N_{s}}{\underset{k=1}{\sum }}{\left({\hat{u}}_{i}\left(\cdot ,\xi^{\left(k\right)}\right)\right)}^{2},$$ (15)

respectively, where *û*_*i*_ = *û*_*i*_ − *μ*(*ũ*_*i*_) is the fluctuation around the mean.

## Results and Discussion

In this section, we report the results obtained when applying computational techniques to numerically solve the proposed diffusion-reaction model, which is an analysis of the behavior of the three mosquito varieties in different scenarios, considering the mendelian (as SM1 [12] mutation, for example) and MCR inheritance [20]. Five situations are considered for numerical experiments. The first one attempts to establish a correlation with a controlled laboratory experience described by Moreira et al. [12]. The following experiments establish different mosquito release scenarios to establish the most effective release strategies. For this purpose, we simulate the population dynamics of each population with different initial conditions. For the mendelian inheritance, no homozygous transgenic mosquito is released, but its population arises naturally from the mating process. In laboratory experiments, it is common to obtain homozygous lines through successive crosses between heterozygous mosquitoes; thus, these mosquitoes are not subject to inbreeding, which can lead to a reduction in fitness [38, 55]. For the MCR inheritance, mosquitoes of the three genotypes will be released, respecting the success rate of the MCR technique in relation to the Mendelian inheritance because of the initial conditions. Indeed, we assume that there is no flow of mosquito populations on the boundary.

In all experiments, we consider that mosquitoes occupy a spatial region *Ī*_*d*_ = [0, 30], measured in km, whose spreading starts from the release of the mosquitoes and has a duration of twelve weeks, *Ī*_*t*_ = [0, 12], corresponding to one season of the year, to avoid seasonal changes in the model parameters.

The finite element mesh adopted consists of three intervals with a uniform mesh sequence. The first and last intervals, given by [0, 10] and [20, 30], respectively, have meshes with a number of nodes (*n* node) equal to 20. In the central region, between ]10, 20[, we consider a more refined mesh with 40 nodes. Linear interpolations (two nodes elements) and quadratic interpolations (three nodes elements) were considered.

As there is no evidence of differences in the flight capacity for wild and transgenic mosquitoes, all mosquitoes have the same probability of dispersal. For this reason, these experiments include the uncertainty in a scenario where the diffusion of wild and transgenic mosquitoes varies randomly in the same range. The random solution of the model was computed by the Monte Carlo method, employing 150 unconditional realizations generated from a uniform distribution in the interval [*s*_*i*_, *s*_*f*_] and considering the diffusion coefficient *κ*_*i*_, *i* = 1, 2, 3 on this interval.

The quality of these realizations was examined for each proposed experiment of the model by comparing the statistical sample’s mean and the cumulative variance of these realizations.

In this paper, we are considering an average sex ratio approximated as 1:1, as is usually done. Estimates on the finite rate of increase for *A*. *stephensi* were obtained by Suleman [56] in laboratory conditions resulting in 1.19 (the population multiplied 1.19 times per day). With this information we can evaluate the weekly finite rate of increase as (1.19)^7^ = 3.38, and then the weekly intrinsic rate of increase is *γ* = ln(3.38) = 1.2. We estimated the instantaneous emergence-rate *ϵ* using the life table from Suleman [56] and expressions from [57], resulting in a rate of 5.1 per week and a death rate *δ*_1_ = (5.1 − 1.2) = 3.9 per week. Obviously, 1.2 is a theoretical potential to increase, and in nature, the population is subject to predation and human action inducing a density-independent mortality, which we will assume to be *δ*_2_ = 0.5 per week. The carrying capacity was assumed to be 2,500 mosquitoes.

The female dispersal (*y*) for *A*. *stephensi* was adjusted by a linear function of the release time (*x*), *y* = 58.741 + 23.731*x*, by Reisen and Aslamkhan [58], resulting in a weekly dispersal of 224.86m. Therefore, the diffusion coefficient can be obtained from $\kappa =\frac{<x^{2}>}{q_{i}t}$, with < *x*^2^ > the mean square displacement and *q*_*i*_ a numerical constante related to dimensionality (*q*_*i*_ = 2, 4, or 6, for 1, 2, or 3-dimensional diffusion, respectively), resulting in *κ* = 0.0253*km*^2^ per week. Thus, it is reasonable to consider the diffusion coefficient varying in the interval [0.01, 0.05].

All parameters used in numerical simulations are shown in Tables 2 and 3.

**Table 2:** Parameters of model estimated from literature data [56, 58].

Parameter	Description	Value
*κ*_*i*_	Diffusion coefficients(Km^2^/week)	[0.01, 0.05]
*ϵ*	Emergence rate to the adult stage (week^−1^)	5.1
*δ*_1_	Density-dependent death rate (week^−1^)	3.9
*δ*_2_	Density-independent death rate (week^−1^)	0.5
*C*	Carrying capacity	2, 500

**Table 3:** Genotypic frequencies considering Mendelian and MCR inheritance.

	Frequency					
	*a*_11_	*a*_12_	*a*_13_	*a*_22_	*a*_23_	*a*_33_
Mendelian	1.0	0.5	0.0	0.25	0.0	0.0
MCR	1.0	0.5	0.0	0.25	0.0	0.0
	*b*_11_	*b*_12_	*b*_13_	*b*_22_	*b*_23_	*b*_33_
Mendelian	0.0	0.5	1.0	0.5	0.5	0.0
MCR	0.0	0.5(1-f)	1.0(1-f)	0.5(1-f)	0.5(1-f)	0.0
	*c*_11_	*c*_12_	*c*_13_	*c*_22_	*c*_23_	*c*_33_
Mendelian	0	0.0	0.0	0.25	0.5	1.0
MCR	0	0.5f	1.0f	0.25+0.5f	0.5+0.5f	1.0

According to the mendelian inheritance, wild mosquitoes *u*_1_ are generated from the mating *u*_1_ × *u*_1_ with frequency *a*_11_ = 1, while *u*_1_ × *u*_2_ with frequency *a*_12_ = 0.5 and *u*_2_ × *u*_2_ with frequency *a*_22_ = 0.25; heterozygous transgenic *u*_2_ are generated from the mating *u*_1_ × *u*_2_ with frequency *b*_12_ = 0.5, *u*_2_ × *u*_2_ with frequency *b*_22_ = 0.5, *u*_2_ × *u*_3_ with frequency *b*_23_ = 0.5 and *u*_1_ × *u*_3_ with frequency *b*_13_ = 1; homozygous transgenic *u*_3_ are generated from the mating *u*_2_ × *u*_2_ with frequency *c*_22_ = 0.25, *u*_2_ × *u*_3_ with frequency *c*_23_ = 0.5, and *u*_3_ × *u*_3_ with frequency *c*_33_ = 1. Considering the MCR inheritance, heterozygous transgenic mosquitoes are converted to homozygous transgenic mosquitoes with a sucess rate f, being 1-f the resistance to this conversion.

The efficiency converting heterozygous *A*. *stephensi* to homozygous transgenic mosquitoes was 98% when obtained from the mating between transgenic males and wild females and 14% when obtained from mating between wild males and transgenic females [20, 21]. In these simulations we will consider *f* = 0.14 (MCR) and *f* = 0 (Mendelian inheritance).

### Simulations using the initial conditions of the laboratory experience

In this experiment, we compared the Hardy-Weinberg frequencies obtained in the laboratory experiment conducted by Moreira et al. [39] with the numerical simulation of the spatial-temporal model (3) considering the spatial dynamics and adopting equivalent initial conditions. In their experiment, the authors confined in a cage equal numbers of wild and heterozygous transgenic mosquitoes, the latter characterized by encoding the anti-Plasmodium SM1 gene in the gut and expressing an enhanced GFP (Green Fluorescent Protein) marker in the eye. After five generations, there was a frequency of 44% of positive GFP and 56% negative GFP, which is consistent with the Hardy-Weinberg equilibrium for allele frequencies *p* = 0.75 and *q* = 0.25; thus, they do not imply fitness load.

The numerical simulation was obtained considering a population composed of 1,000 wild and 1,000 heterozygous transgenic mosquitoes, homogeneously spread over 4 km, according to the following initial condition:

$$\begin{array}{c} \begin{array}{c} u_{1}\left(x,0\right)=u_{2}\left(x,0\right)=\{\begin{array}{c} 250\;\;if\;13\leq x\leq 17,\\ 0\;\;if\;0\leq x<13\;and\;17<x\leq 30 \end{array}\\ u_{3}\left(x,0\right)=0. \end{array} \end{array}$$ (16)

**Fig 1:**
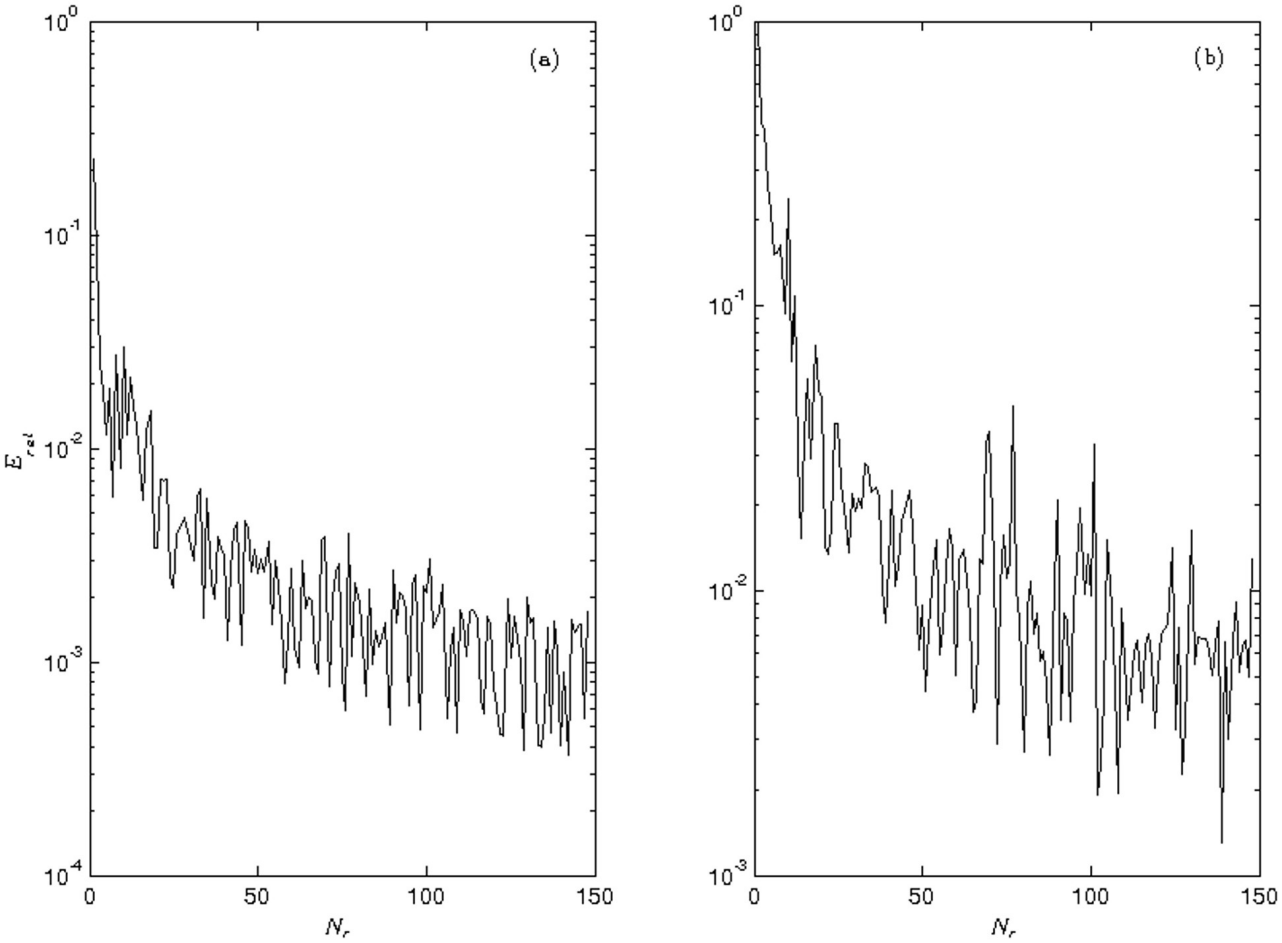
Statistical convergence of the first and second moment of the wild mosquito population. Relative error: (a) Mean and (b) Variance.

This result means that the transgene frequency in the parental population was *q* = 1/4, as in the laboratory experiment, and invariant throughout the 4 km, ensuring that encounters between mosquito varieties occurred with the same probability and randomness. To check the quality of the realizations, we computed the results of various samples in the last step of time and calculated the relative error of the mean and variance of wild mosquitoes defined as

$$\begin{array}{c} E_{rel}\left(N_{s}\right)=\frac{∥m_{u_{1}}\left(N_{s}\right)-m_{u_{1}}\left(N_{s}+1\right)∥}{∥m_{u_{1}}\left(N_{s}+1\right)∥}, \end{array}$$ (17)

where $m_{u_{1}}\left(N_{s}\right)$ denotes the mean or variance of *N*_*s*_ samples generated by the target solution which was computed on *L*^2^-norm. Once the relative error of the mean and variance for the *N*_*r*_ realizations were computed, we chose the target solution with 150 realizations since the fluctuation of the relative error in both moments converged to values close to or below 1% (see Fig 1). Similar results were obtained for the transgenic populations, and therefore, they were omitted from the text in all other experiments.

**Fig 2:**
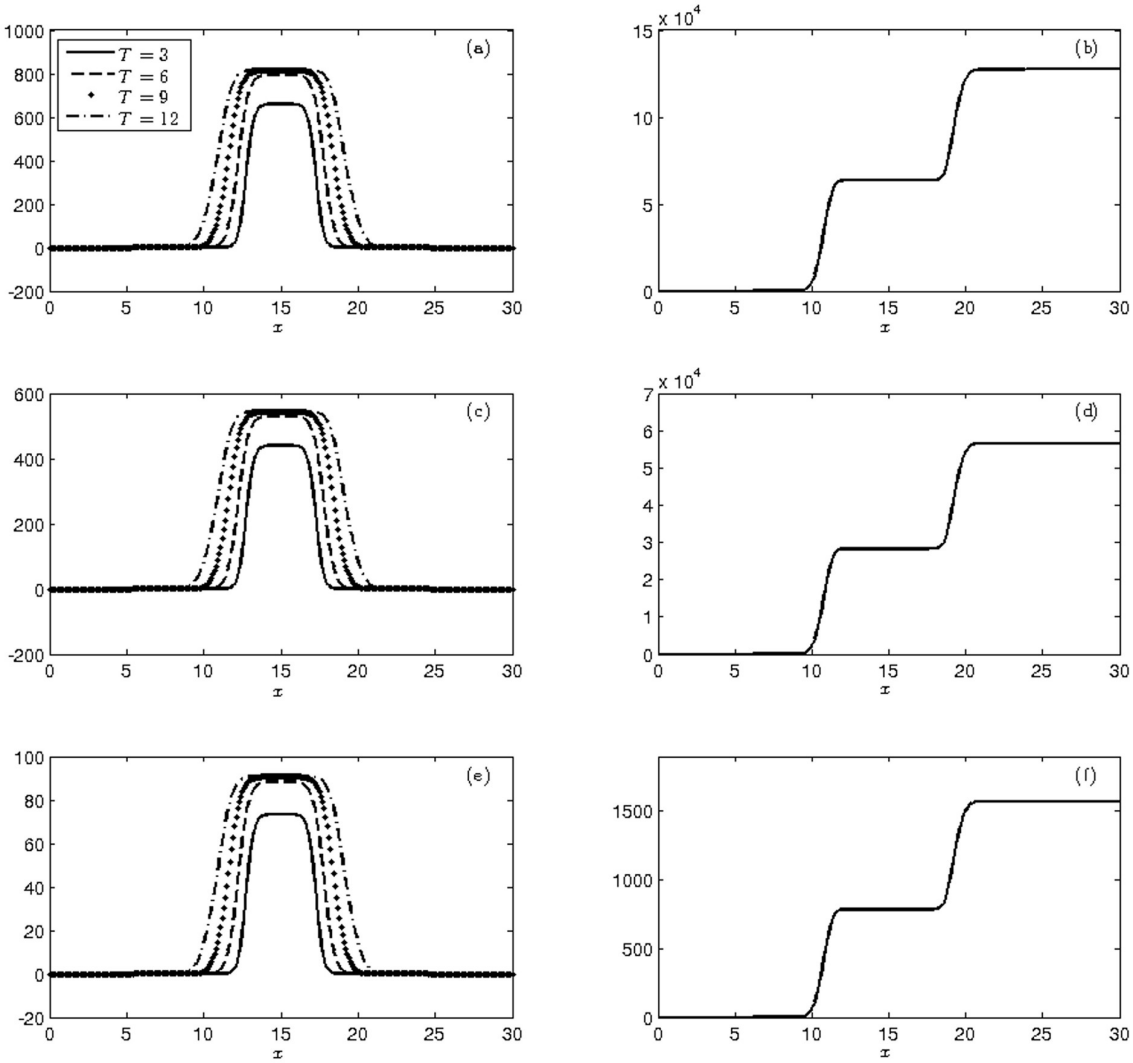
Display of the moments at times T = 3, 6, 9, 12 weeks of (a)-(c)-(e) mean and (b)-(d)-(f) cumulative variance of the population of wild (a-b), heterozygous (c-d) and homozygous transgenic mosquitoes (e-f) with initial conditions (16).

In Fig 2 we plotted the behavior of the mean and cumulative variance of the three mosquito varieties. The average number of mosquitoes obtained at the end of each quarter, represented by mean *μ*^*T*^(·), that resulted from the integration of the obtained solution into the *Ī*_*d*_ domain using the trapezoidal rule is presented in Table 4. In the second moment, we used the cumulative variance in order to better visualize the discrepancy between the average number of mosquitoes for each variety obtained from the fixed and random diffusion parameters.

**Table 4:** Total populations of the corresponding estimated values of the mean at T = 3, 6, 9, 12, using Monte Carlo simulations and initial conditions (16).

Mean	Time			
	*T* = 3	*T* = 6	*T* = 9	*T* = 12
*μ*^*T*^(*u*_1_)	2.9986e+03	4.4260e+03	5.6266e+03	6.8316e+03
*μ*^*T*^(*u*_2_)	1.9990e+03	2.9507e+03	3.7511e+03	4.5544e+03
*μ*^*T*^(*u*_3_)	333.1725	491.7788	625.1760	759.0633

Table 4 shows that the means obtained at the end of the integration process correspond to approximately 56% wild mosquitoes and 44% transgenic (heterozygous plus homozygous) mosquitoes, which is in agreement with the experiment described in [39]. Moreover, these proportions are in accordance with the Hardy-Weinberg equilibrium for *ŭ*_1_(0) = *ŭ*_2_(0) = 0.5 and *ŭ*_3_(0) = 0 (see S1 Appendix), which are equivalent to the initial conditions (16).

We also note that the cumulative variance in all experiments starts very small due to the strong influence of the deterministic initial data. As time goes by, the profile grows with a higher uncertainty in that there is a growth near 10 km, and then, the population stabilizes between 10 km and 20 km, expanding again after that limit and then stabilizing. At the end of the simulation process, which comprises a 12-week period, the cumulative variance of the total number of mosquitoes was approximately 68% wild mosquitos and 32% transgenic mosquitoes.

### Simulations involving effective study of mosquito release strategies

In the following experiments, we describe scenarios obtained from the numerical simulation of model (3) for different initial configurations of mosquito distribution in the domain. In all cases, we consider an initial population composed of 2,000 wild, 1,000 heterozygous and no homozygous mosquitos for the mendelian case and 2,000 wild, 860 heterozygous and 140 homozygous mosquitoes for the MCR case, distributed over 30 km in different ways. These values were assumed considering that 14% of the heterozygous mosquitoes became homozygous due to the MCR technology. The analysis of these simulations gives us information about the best way to release transgenic mosquitoes, aiming to increase in this population the frequency of the gene that interferes with the development of the protozoan. For this simulation, we assumed that wild mosquitoes are in a region classified as one of high incidence and that they spread to a region of lower concentration within a domain, being contained due to the interaction with transgenic mosquitoes, which spread in the same way.

**Experiment 1-Mendelian**: In this experiment, we consider an initial population distributed over 30 km in the following way:

$$\begin{array}{c} \begin{array}{c} u_{1}\left(x,0\right)=\{\begin{array}{c} 500\;\;if\;13\leq x\leq 17,\\ 0\;\;if\;0\leq x<13\;and\;17<x\leq 30, \end{array}\\ u_{2}\left(x,0\right)=\{\begin{array}{c} 250\;\;if\;13\leq x\leq 17,\\ 0\;\;if\;0\leq x<13\;and\;17<x\leq 30, \end{array}\\ u_{3}\left(x,0\right)=0. \end{array} \end{array}$$ (18)

These conditions characterize a release in which 2,000 wild mosquitoes are homogeneously distributed in nature, strewn over a 4km strip. In this simulation, 1,000 heterozygous transgenic mosquitoes are introduced, maintaining the same uniformity characteristic of wild mosquitoes. This distribution characterizes a transgene frequency in the parental population of *q* = 1/6, along this strip. This allele frequency (*p* = 5/6, *q* = 1/6) leads to a Hardy–Weinberg equilibrium of *f*(*w*, *w*) = 0.6944, *f*(*w*, *g*) = 0.2778 and *f*(*g*, *g*) = 0.0278.

**Fig 3:**
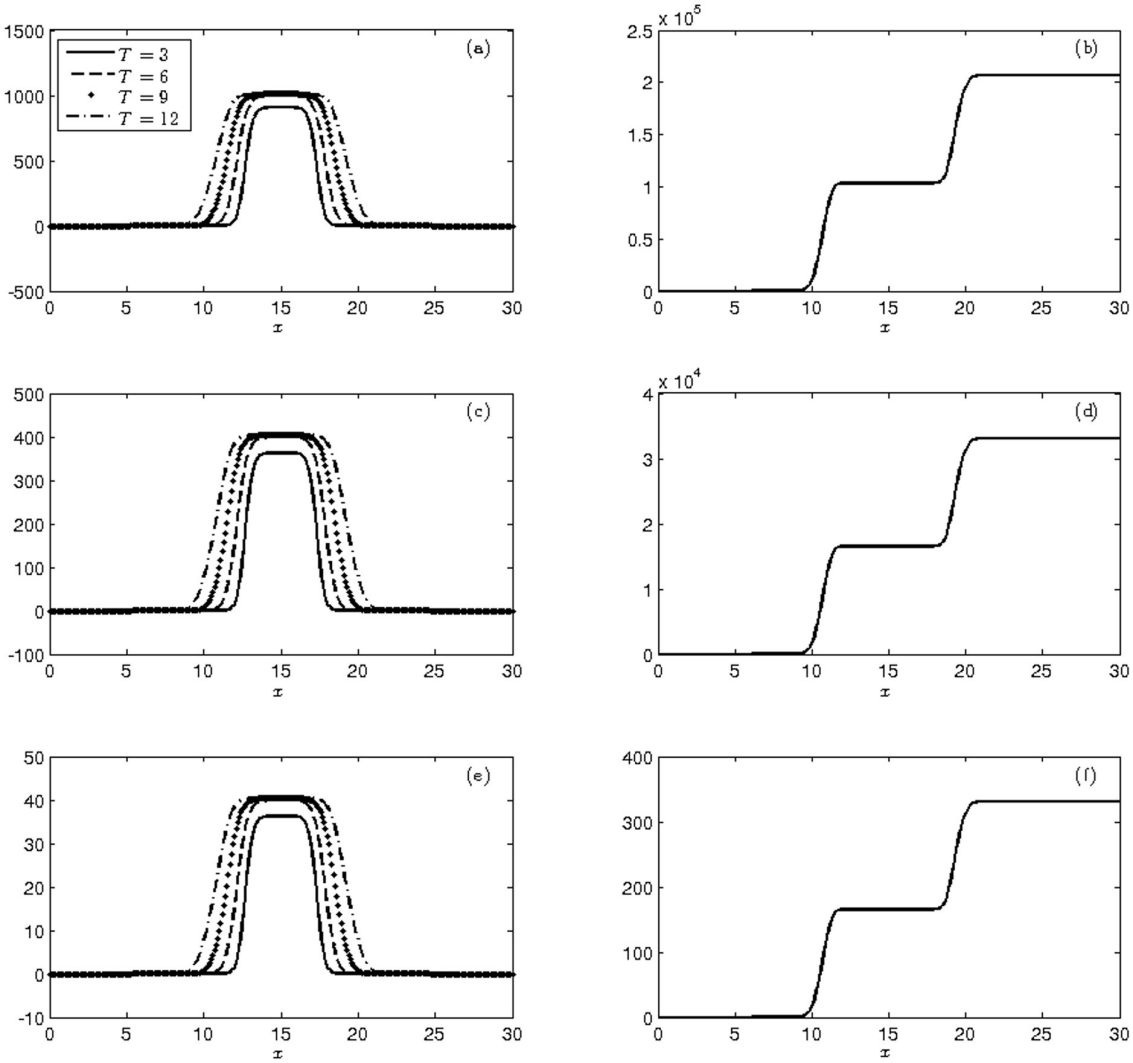
Display of the moments at times T = 3, 6, 9, 12 weeks of (a)-(c)-(e) mean and (b)-(d)-(f) cumulative variance of the populations of wild (a-b), heterozygous (c-d) and homozygous transgenic mosquitoes (e-f) with initial conditions (18).

Fig 3 illustrates the means and cumulative variances of wild, heterozygous and homozygous transgenic mosquitoes. As the release form of this experiment is similar to the previously discussed laboratory experience, it is expected that the behavior of the graphs in these figures have the same evolution at the statistical moments found in Fig 2. The experimental results are summarized and listed in Table 5.

**Table 5:** Total populations of the corresponding estimated values of the mean at T = 3, 6, 9, 12, using Monte Carlo simulations and initial conditions (18).

Mean	Time			
	*T* = 3	*T* = 6	*T* = 9	*T* = 12
*μ*^*T*^(*u*_1_)	4.1832e+03	5.6913e+03	7.1280e+03	8.6228e+03
*μ*^*T*^(*u*_2_)	1.6733e+03	2.2765e+03	2.8512e+03	3.4491e+03
*μ*^*T*^(*u*_3_)	167.3289	227.6532	285.1201	344.9122

The increase in wild mosquitoes in the initial conditions passed from the ratio of 1:1 in (16) to 2:1 in (18) led, at the end of simulation, to proportions of 69.44% wild, 27.78% heterozygous and 2.78% homozygous transgenic mosquitoes, with a frequency of transgenic alleles of q = 0.167. These values are expected and consistent with the Hardy-Weinberg equilibrium for the initial conditions of proportions *ŭ*_1_(0) = 2/3, *ŭ*_2_(0) = 1/3 and *ŭ*_3_ (0) = 0, which are equivalent to the initial conditions in (18). This initial distribution consequently contributed to the cumulative variance growth of wild mosquitoes and the decrease in transgenic mosquitoes.

**Experiment 1-MCR**: In this experiment, we consider an initial population distributed over 30 km in the following way:

$$\begin{array}{c} \begin{array}{c} u_{1}\left(x,0\right)=\{\begin{array}{c} 500\;\;if\;13\leq x\leq 17,\\ 0\;\;if\;0\leq x<13\;and\;17<x\leq 30, \end{array}\\ u_{2}\left(x,0\right)=\{\begin{array}{c} 215\;\;if\;13\leq x\leq 17,\\ 0\;\;if\;0\leq x<13\;and\;17<x\leq 30, \end{array}\\ u_{3}\left(x,0\right)=\{\begin{array}{c} 35\;\;if\;13\leq x\leq 17,\\ 0\;\;if\;0\leq x<13\;and\;17<x\leq 30. \end{array} \end{array} \end{array}$$ (19)

**Fig 4:**
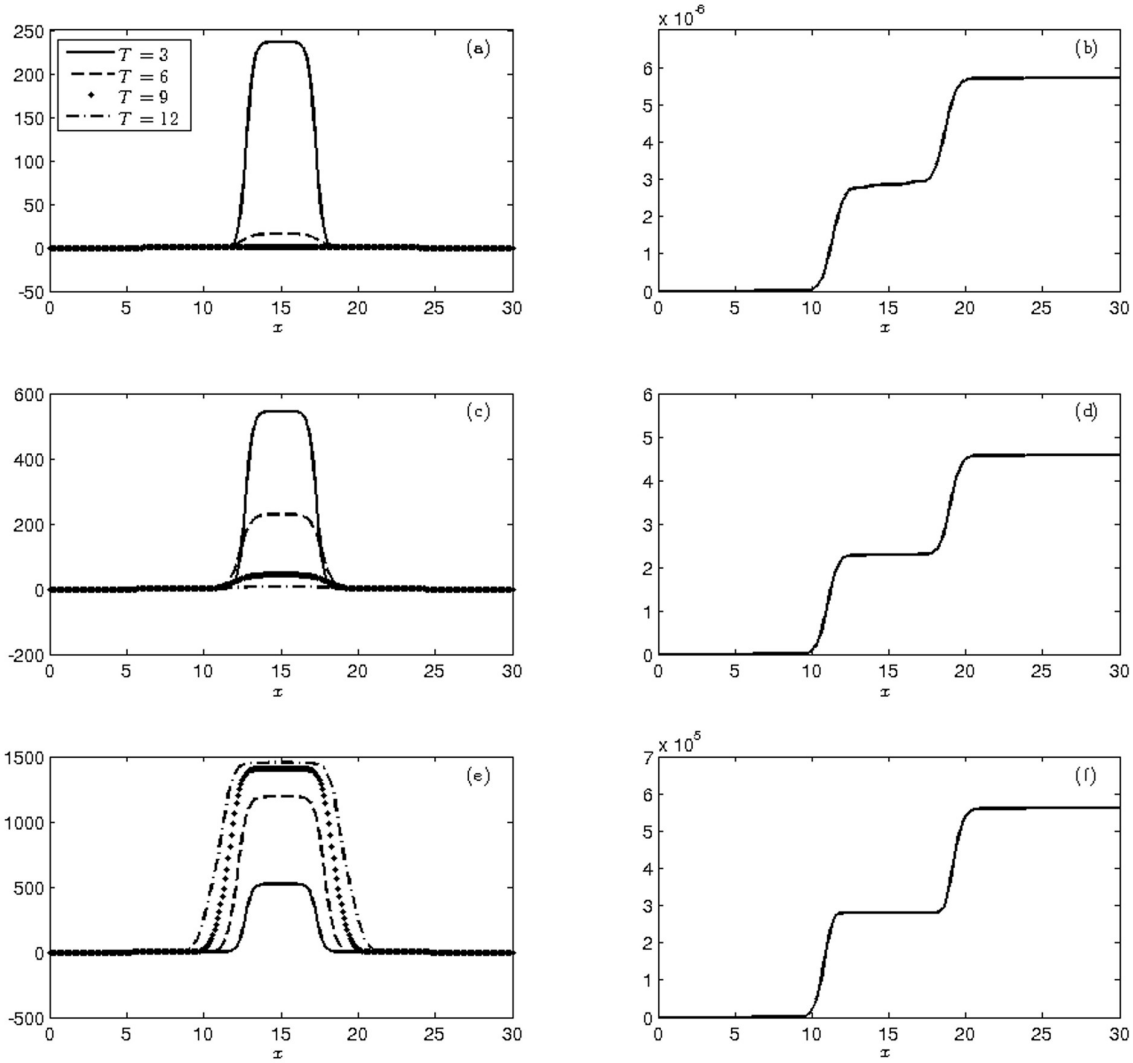
Display of the moments at times T = 3, 6, 9, 12 weeks of (a)-(c)-(e) mean and (b)-(d)-(f) cumulative variance of the populations of wild (a-b), heterozygous (c-d) and homozygous transgenic mosquitoes (e-f) with initial conditions (19).

Under these conditions, 2,000 wild mosquitoes are homogeneously distributed in nature, strewn over a 4 km strip. In the same region, 860 heterozygous and 140 homozygous transgenic mosquitoes are introduced maintaining the same uniformity characteristic of wild mosquitoes. The frequency of transgenic alleles in the parental population is *q* = 0.19. The evolution of the three population for 12 weeks can be seen in Fig 4. From the mean, we can see a high population growth of transgenic mosquitoes, pre-empting the homozygous mosquitoes.

Table 6 shows the total populations at 3, 6, 9 and 12 weeks. After the final week, 99.62% of population was composed of homozygous transgenic mosquitoes, and 0.37% was composed of heterozygous mosquitoes, implying a frequency of transgenic alleles of q = 0.998. The proportion of the wild type of mosquito was not relevant.

**Table 6:** Total populations of the corresponding estimated values of the mean at T = 3, 6, 9, 12, using Monte Carlo simulations and initial conditions (19).

Mean	Time			
	*T* = 3	*T* = 6	*T* = 9	*T* = 12
*μ*^*T*^(*u*_1_)	1.0439e+03	76.9052	2.5391	0.0690
*μ*^*T*^(*u*_2_)	2.5367e+03	1.2388e+03	273.2076	49.7368
*μ*^*T*^(*u*_3_)	2.5418e+03	7.1854e+03	1.0538e+04	1.3175e+04

**Experiment 2-Mendelian**: In this experiment, we simulate the mosquitoes’ release considering the same initial distribution of wild mosquitoes as the previous experiment; however, we adopt the release of 1,000 heterozygous transgenic mosquitoes in a concentrated way, restricted to a small region of 1 km. This situation is described by initial conditions:

$$\begin{array}{c} \begin{array}{c} u_{1}\left(x,0\right)=\{\begin{array}{c} 500\;\;if\;13\leq x\leq 17,\\ 0\;\;if\;0\leq x<13\;and\;17<x\leq 30, \end{array}\\ u_{2}\left(x,0\right)=\{\begin{array}{c} 1,000\;\;if\;14.5\leq x\leq 15.5,\\ 0\;\;\;if\;0\leq x<14.5\;and\;15.5<x\leq 30, \end{array}\\ u_{3}\left(x,0\right)=0. \end{array} \end{array}$$ (20)

Here, populations are not evenly distributed in the same space, and therefore, mating occurs naturally among the nearest mosquito varieties. Due to this distance-induced preference, mating is not random, and the Hardy-Weinberg equilibrium cannot be verified. This deviation of randomness by geographic dispersion will occur in all experiments hereafter.

**Fig 5:**
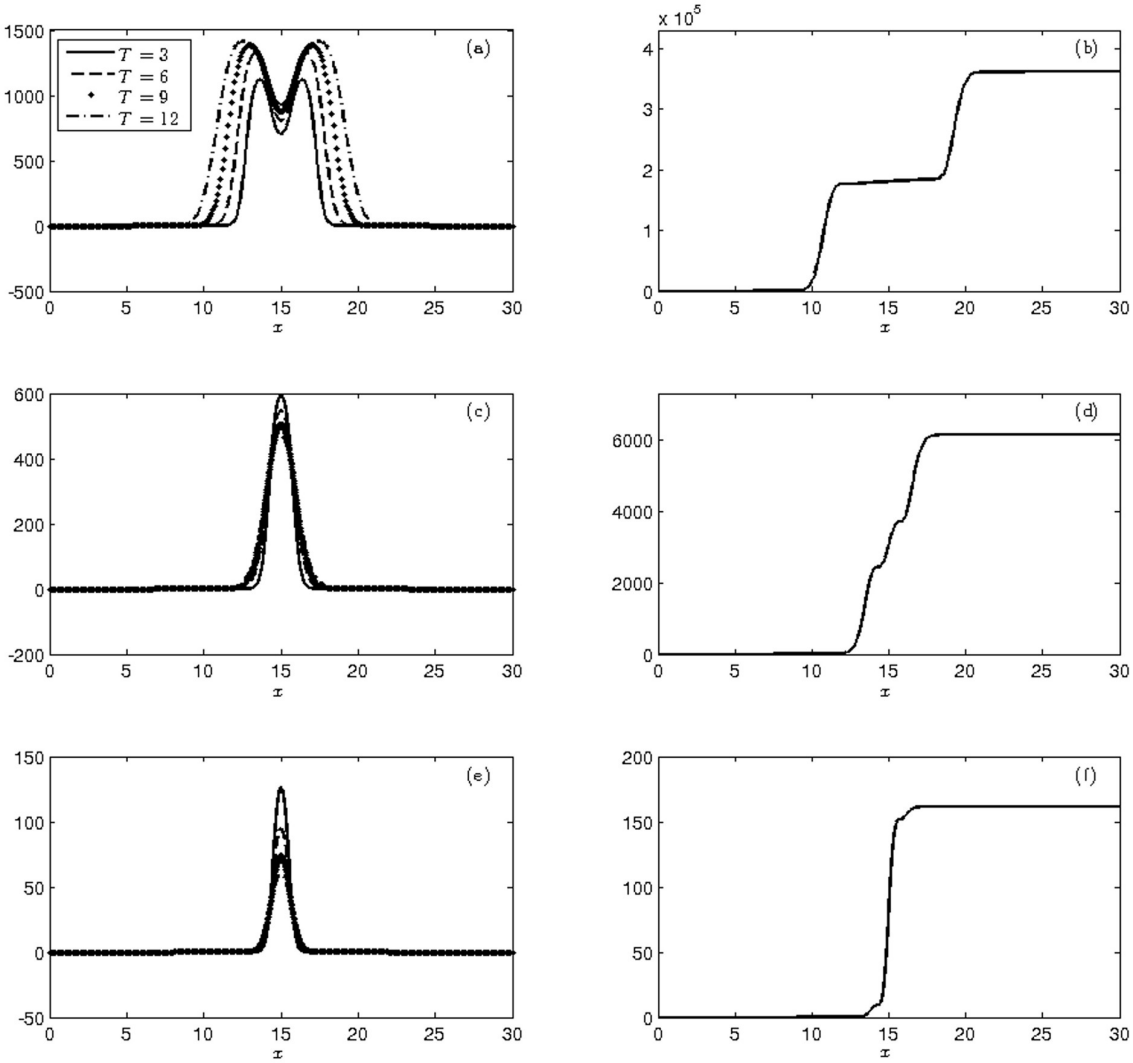
Display of the moments at times T = 3, 6, 9, 12 weeks of (a)-(c)-(e) mean and (b)-(d)-(f) cumulative variance of the populations of wild (a-b), heterozygous (c-d) and homozygous transgenic mosquitoes (e-f) with initial conditions (20).

Numerical simulations corresponding to the first and second moments using initial conditions (20) are shown in Fig 5. We can also observe that the growth in the cumulative variance is more pronounced in heterozygous and homozygous transgenic mosquitoes.

In the early part of the simulation, in intervals [13, 14.5] and [15.5, 17] almost all the mating and generating are by wild mosquitoes; therefore, wild-type mosquitoes cause the increase in the mean of this variety in these intervals. In the region [14.5, 15.5], there are twice as many transgenic mosquitoes compared to wild ones, with the mating generating the three varieties of mosquitoes. Gradually peaks and valleys tend soften and merge. After twelve weeks, we obtain from Table 7 proportions of 89.63% wild, 9.57% heterozygous and 0.8% homozygous transgenic mosquitoes, with a final transgene frequency of *q* = 0.056, much lower than the Hardy-Weinberg frequency of *q* = 0.167 obtained in Experiment 1—Mendelian. Comparing these values obtained using (20) with those obtained from the initial conditions (18), we can observe an increase in wild mosquitoes and a decrease in transgenic mosquitoes due to the method of release. Releasing transgenic mosquitoes in a more restricted region induced 27.36% more wild mosquitoes and 66% and 71.63% fewer heterozygous and homozygous transgenic mosquitoes, respectively, than in a homogeneous release process over 4 km.

**Table 7:** Total populations of the corresponding estimated values of the mean at T = 3, 6, 9, 12, using Monte Carlo simulations and initial conditions (20).

Mean	Time			
	*T* = 3	*T* = 6	*T* = 9	*T* = 12
*μ*^*T*^(*u*_1_)	4.6029e+03	6.7495e+03	8.8277e+03	1.0982e+04
*μ*^*T*^(*u*_2_)	952.7485	1.0881e+03	1.1433e+03	1.1727e+03
*μ*^*T*^(*u*_3_)	140.8560	123.4126	108.7569	97.8511

**Experiment 2-MCR**: In this experiment, we adopted the same release strategy as the mendelian case, but considering initial conditions for MCR:

$$\begin{array}{c} \begin{array}{c} u_{1}\left(x,0\right)=\{\begin{array}{c} 500\;\;if\;13\leq x\leq 17,\\ 0\;\;if\;0\leq x<13\;and\;17<x\leq 30, \end{array}\\ u_{2}\left(x,0\right)=\{\begin{array}{c} 860\;\;if\;14.5\leq x\leq 15.5,\\ 0\;\;\;if\;0\leq x<14.5\;and\;15.5<x\leq 30, \end{array}\\ u_{3}\left(x,0\right)=\{\begin{array}{c} 140\;\;if\;14.5\leq x\leq 15.5,\\ 0\;\;\;if\;0\leq x<14.5\;and\;15.5<x\leq 30. \end{array} \end{array} \end{array}$$ (21)

**Fig 6:**
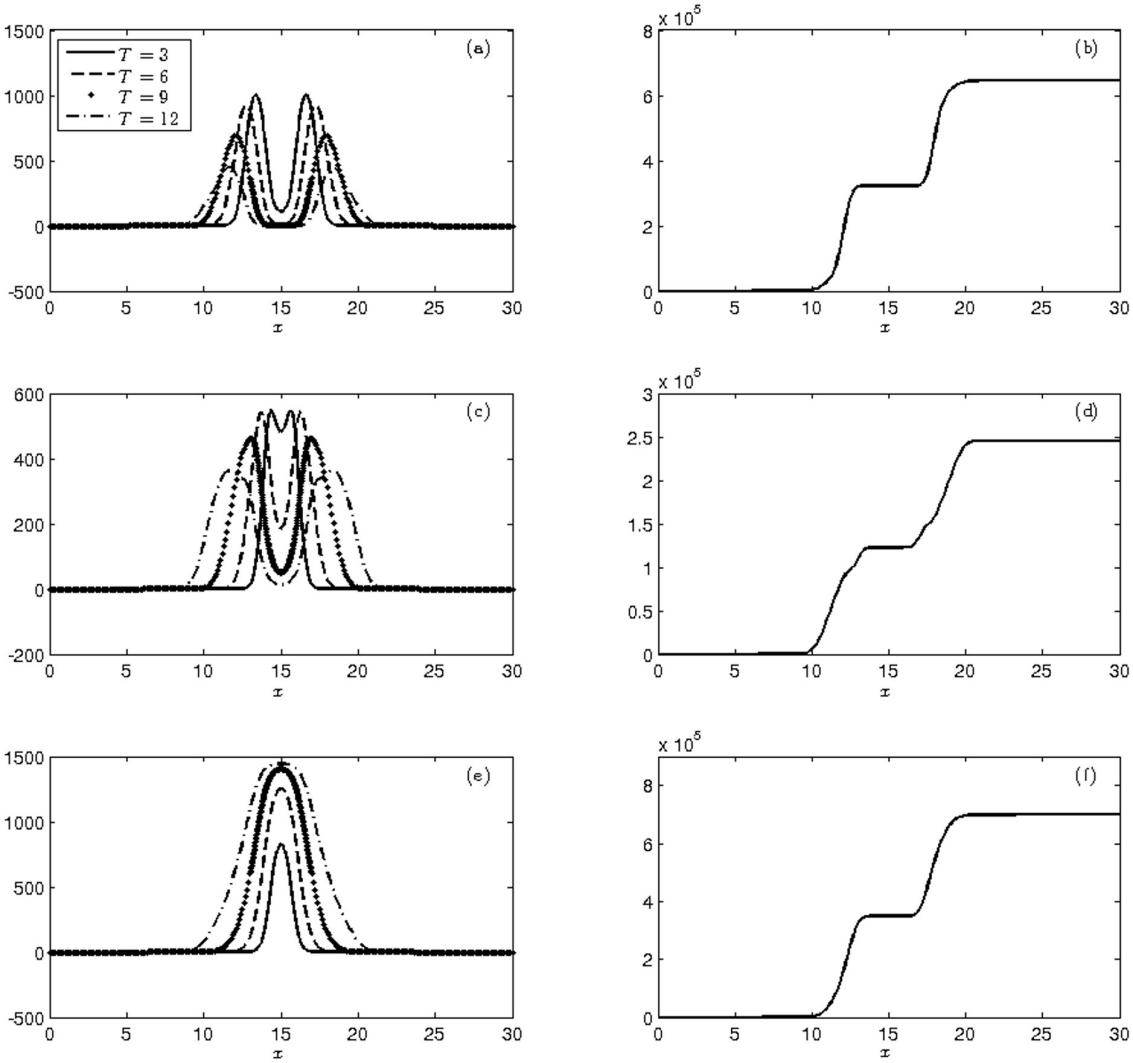
Display of the moments at times T = 3, 6, 9, 12 weeks of (a)-(c)-(e) mean and (b)-(d)-(f) cumulative variance of the populations of wild (a-b), heterozygous (c-d) and homozygous transgenic mosquitoes (e-f) with initial conditions (21).

Fig 6 shows the mean and cumulative variance of the three populations at four moments. Due to initial conditions, there is a strong decay of the wild mosquito population at the center of the domain, contrasting with the homozygous mosquito population which continues to grow as time progresses. Furthermore, the population of heterozygous mosquitoes has a strong growth in the intervals [9.5, 14.5] and [15.5, 21]. The cumulative variances indicates a balance in the population growth for both trangenic and wild mosquitoes.

Finally, 12,821 mosquitoes were accounted. The final proportions were 16.02% wild type, 18.23% heterozygous and 65.75% homozygous transgenic, as shown in Table 8. A release of transgenic mosquitoes according to Experiment 2 (Mendelian or MCR) is the more intuitive manner of inserting transgenic mosquitoes into the environment; however, this method is the least effective. Despite this result, the experiment showed that the MCR is efficient even in this situation, leading to a final frequency of transgenic alleles of q = 0.75.

**Table 8:** Total populations of the corresponding estimated values of the mean at T = 3, 6, 9, 12, using Monte Carlo simulations and initial conditions (21).

Mean	Time			
	*T* = 3	*T* = 6	*T* = 9	*T* = 12
*μ*^*T*^(*u*_1_)	3.1396e+03	3.2292e+03	2.8612e+03	2.0545e+03
*μ*^*T*^(*u*_2_)	1.3376e+03	1.8159e+03	2.1513e+03	2.3371e+03
*μ*^*T*^(*u*_3_)	1.2706e+03	3.1068e+03	5.4399e+03	8.4295e+03

**Experiment 3-Mendelian**: In this experiment, we investigate the dynamics of the three species of mosquitoes, considering a release region of the heterozygous transgenic mosquitoes larger than the natural region of the wild-type mosquitoes. For this, we consider an initial condition in which 2,000 wild mosquitoes are found in a 4 km region, and 1,000 heterozygous transgenic mosquitoes are evenly distributed in a 6 km region. This initial configuration is given by the following conditions:

$$\begin{array}{c} \begin{array}{c} u_{1}\left(x,0\right)=\{\begin{array}{c} 500\;\;if\;13\leq x\leq 17,\\ 0\;\;if\;0\leq x<13\;and\;17<x\leq 30, \end{array}\\ u_{2}\left(x,0\right)=\{\begin{array}{c} 166.67\;\;if\;12\leq x\leq 18,\\ 0\;\;if\;0\leq x<12\;and\;18<x\leq 30, \end{array}\\ u_{3}\left(x,0\right)=0. \end{array} \end{array}$$ (22)

To begin, only heterozygous transgenic mosquitoes are over the intervals [12, 13] and [17, 18]. Mating between mosquitoes of this population generates 50% heterozygous, twice the other varieties; because of this, there is an increase in heterozygous transgenic mosquitoes in these regions. Mating between heterozygous mosquitoes also generates more homozygous transgenic mosquitoes than mating between mosquitoes of a mixed population composed of the wild type and the heterozygous; thus, the population of homozygous transgenic mosquitoes is also increased in positions [12, 13] and [17, 18].

**Fig 7:**
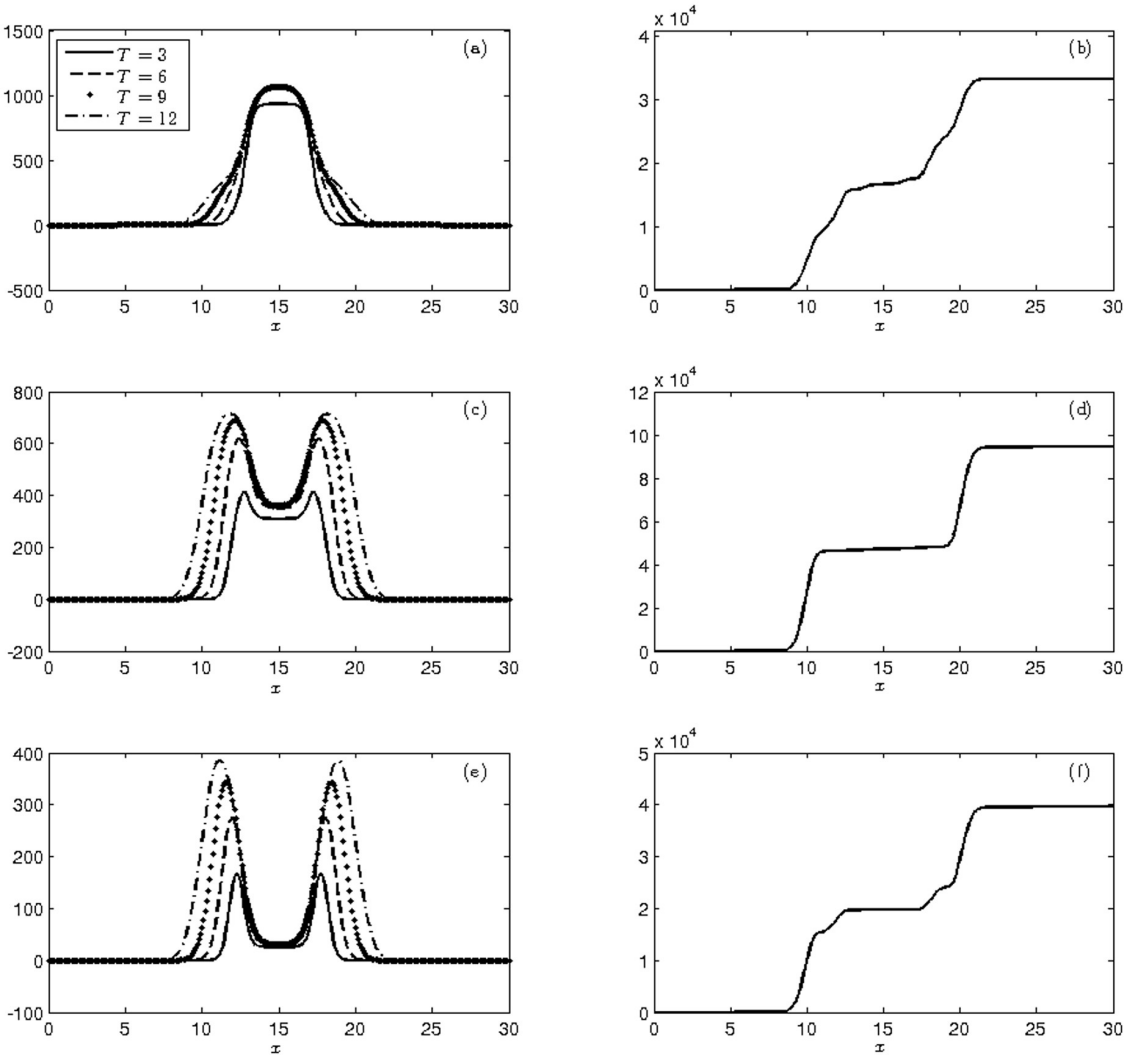
Display of the moments at times T = 3, 6, 9, 12 weeks of (a)-(c)-(e) mean and (b)-(d)-(f) cumulative variance of populations of wild (a-b), heterozygous (c-d) and homozygous transgenic mosquitoes (e-f) with initial conditions (22).

This dynamic is shown in Fig 7, which illustrates the mean and cumulative variance of the three populations at four moments.

Table 9 shows that the growth of wild mosquitoes is lower than that presented in previous experiments, comprising 43.7% of total population. On the other hand, the numbers of heterozygous and homozygous transgenic mosquitoes showed a very significant growth, reaching 40% and 16.3% of the total population, respectively. Consequently, the population growth rate in the cumulative variation is higher for transgenic mosquitoes. Therefore, the release of transgenic mosquitoes that extrapolate the focus region of the wild-type mosquitoes presents the best way of transgenic mosquito insertion into the total population, resulting in a frequency of the transgene alleles of q = 0.363, the highest value obtained from Mendelian genetics.

**Table 9:** Total populations of the corresponding estimated values of the mean at T = 3, 6, 9, 12, using Monte Carlo simulations and initial conditions (22).

Mean	Time			
	*T* = 3	*T* = 6	*T* = 9	*T* = 12
*μ*^*T*^(*u*_1_)	4.2924e+03	5.4036e+03	5.9782e+03	6.4293e+03
*μ*^*T*^(*u*_2_)	2.2320e+03	3.5904e+03	4.8033e+03	5.9805e+03
*μ*^*T*^(*u*_3_)	506.5637	1.0281e+03	1.6238e+03	2.2700e+03

**Fig 8:**
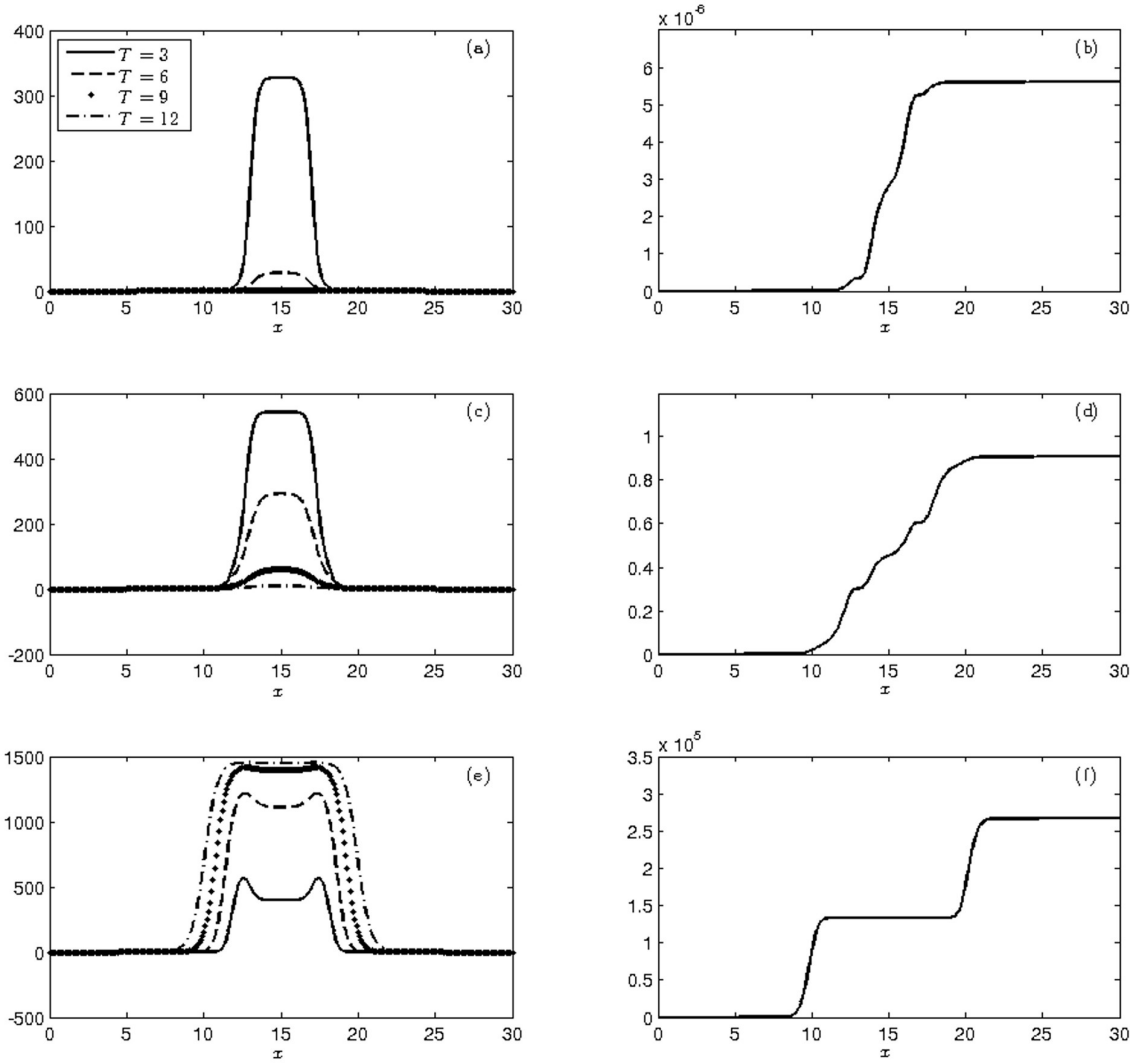
Display of the moments at times T = 3, 6, 9, 12 weeks of (a)-(c)-(e) mean and (b)-(d)-(f) cumulative variance of populations of wild (a-b), heterozygous (c-d) and homozygous transgenic mosquitoes (e-f) with initial conditions (23).

**Experiment 3-MCR**: This initial configuration is given by the following conditions:

$$\begin{array}{c} \begin{array}{c} u_{1}\left(x,0\right)=\{\begin{array}{c} 500\;\;if\;13\leq x\leq 17,\\ 0\;\;if\;0\leq x<13\;and\;17<x\leq 30, \end{array}\\ u_{2}\left(x,0\right)=\{\begin{array}{c} 143.33\;\;if\;12\leq x\leq 18,\\ 0\;\;if\;0\leq x<12\;and\;18<x\leq 30, \end{array}\\ u_{3}\left(x,0\right)=\{\begin{array}{c} 23.33\;\;if\;12\leq x\leq 18,\\ 0\;\;if\;0\leq x<12\;and\;18<x\leq 30. \end{array} \end{array} \end{array}$$ (23)

considering the same pattern of initial distribution as the previous experiment, but with values according to the MCR. We plot the mean and cumulative variance corresponding to these initial conditions (see Fig 8).

Fig 8 exhibits the dominance of the homozygous mosquito population and the decay of the other populations. This is reflected in the cumulative variance that retains a strong variability in homozygous mosquitoes while the other population has an almost negligible variability.

Table 10 shows that of the 14,434 mosquitoes obtained at the end of the 12 weeks, 99.7% were homozygous transgenic and 0.3% were heterozygous, indicating that the transgenic allele is the most frequent, reaching a frequency of q = 0.9985.

**Table 10:** Total populations of the corresponding estimated values of the mean at T = 3, 6, 9, 12, using Monte Carlo simulations and initial conditions (22).

Mean	Time			
	*T* = 3	*T* = 6	*T* = 9	*T* = 12
*μ*^*T*^(*u*_1_)	1.2716e+03	97.0692	2.9900	0.0753
*μ*^*T*^(*u*_2_)	2.6539e+03	1.3061e+03	264.8893	44.2056
*μ*^*T*^(*u*_3_)	3.0904e+03	8.5488e+03	1.1985e+04	1.4390e+04

## Conclusion

In this paper, we present the development of a new mathematical model to describe the interaction and spreading of wild and transgenic mosquitoes taking into account the zygosity, considering all populations in absolute numbers, and admitting random diffusion coefficients. These characteristics make the model description more realistic, ensuring the existence of all possible phenotypes, preserving the parameters that do not exist in dimensionless form and allowing for the mosquitos a nonuniform displacement.

The dynamic system was developed to preserve the peculiarities of the species and avoid an overlap of individuals when the transgenics are inserted, respecting the environmental support capacity. The terms describing mating and competition imply the nonlinearities of this system, which are characteristic of the great majority of population dynamics models. The diffusion term, based on Fick’s law, describes a symmetrical spread of the mosquito population with a random dispersal.

The solution of the proposed problem was obtained using the operator splitting method to decouple the diffusion-reaction system into two subproblems, in which the diffusive problem was solved employing the finite element method and the reactive problem using the fourth order Runge-Kutta method.

The results shown are consistent with the expected behavior, indicating a constant presence of transgenic mosquitoes after entering the ecosystem and reducing periodic insertion costs, as is the case with sterile mosquitoes. Numerical simulations provide guidelines, indicating a higher efficiency when the release of the transgenics is done in a broader coverage. On the other hand, the worst strategy is the release of transgenic mosquitoes concentrated in a small single region, allowing for the wild type to mate and breed, while the contact with transgenics is not yet accomplished. Without the imposition of transgenics’ superiority, the total elimination of wild-type mosquitoes is impossible to achieve, since they are also obtained from the mating between heterozygous transgenics. It is worth mentioning that the extinction of a species should not be the main objective; it is sufficient that the population of wild-type mosquitoes is reduced to levels that do not harm human health, and this is possible for both classical Mendelian genetics and MCR. However, the frequency of transgenic alleles for the MCR is much higher than that for Mendelian genetics, MCR being the most efficient technique in all the experiments presented.

The impact of reducing infectious diseases transmitted by mosquitoes can be studied by coupling the model proposed in this paper with an appropriate epidemiological dynamic. By identifying an effective breeding site, it is possible to plan an optimal strategy for the release of transgenic mosquitoes, optimizing the allocation of scarce resources. Thus, the entry of genetically modified individuals into the ecosystem can be a viable alternative to disease control, as long as they are able to interact with the wild mosquitoes and have at least the same survival capacity in the environmental field.

This work is an initial proposal that opens possibilities for future investigations. Initial conditions most appropriate to reality need to be tested in order to obtain a strategy for the release of transgenic insects into the environment. Factors such as seasonality, temperature, winds, rainfall, and fitness cost can be introduced into this model, or in a two-dimensional version of it, making it even more realistic. The choice of the finite element method to solve the diffusive problem is intended to facilitate some of these future steps.

## Supporting information

S1 Appendix (PDF)
