# Supplementary material for: Modeling the spreading and interaction between wild and transgenic mosquitoes with a random dispersal
Source: PLoS One. 2018 Oct 31;13(10):e0205879. doi: 10.1371/journal.pone.0205879 (PMC6209212; doi:10.1371/journal.pone.0205879)
Supplement: S1 Appendix — (PDF) [file pone.0205879.s001.pdf]

Appendix

Existence of positive solutions and stability results of the model without mosquito dispersal

In the following, we present some results which guarantee that the model governed by system

$$\begin{cases} \dfrac{du_1}{dt} = \left(\dfrac{\epsilon}{\sum u_i} - \dfrac{\gamma}{C}\right) \sum\sum a_{ij} u_i u_j - \delta u_1, \\ \dfrac{du_2}{dt} = \left(\dfrac{\epsilon}{\sum u_i} - \dfrac{\gamma}{C}\right) \sum\sum b_{ij} u_i u_j - \delta u_2, \\ \dfrac{du_3}{dt} = \left(\dfrac{\epsilon}{\sum u_i} - \dfrac{\gamma}{C}\right) \sum\sum c_{ij} u_i u_j - \delta u_3, \end{cases} \tag{1}$$

with initial conditions

$$u_i(0) = \overline{u}_i, \tag{2}$$

is biologically and mathematically well-posed in a feasible region $\mathscr{D}$ given by:

$$\mathscr{D} = \left\{ (u_1, u_2, u_3) \in I\!R^3 / \ u_1 \geq 0, u_2 \geq 0, u_3 \geq 0, \sum u_i \leq N^* \right\}. \tag{3}$$

Theorem 1. *Considering the system (1), there is a unique solution set $\{u_1(t), u_2(t), u_3(t)\}$ of system (1) with nonnegative initial conditions (2) in the feasible domain $\mathscr{D}$, that remains nonnegative in $\mathscr{D}$ for all $t \geq 0$.*

Proof. The functions of the right hand side of the system (1) are continuous with continuous derivatives in $\mathscr{D}$, therefore exists a unique solution for all time $t \geq 0$. It remains to be shown that $\mathscr{D}$ is forward-invariant. We can see that

if $u_1 = 0$ then $\dfrac{du_1}{dt} = \left(\dfrac{\epsilon}{u_2 + u_3} - \dfrac{\gamma}{C}\right) \sum_{i \neq 1} \sum a_{ij} u_i u_j \geq 0;$

if $u_2 = 0$ then $\dfrac{du_2}{dt} = \left(\dfrac{\epsilon}{u_1 + u_3} - \dfrac{\gamma}{C}\right) \sum_{i \neq 2} \sum b_{ij} u_i u_j \geq 0;$

if $u_3 = 0$ then $\dfrac{du_3}{dt} = \left(\dfrac{\epsilon}{u_1+u_2} - \dfrac{\gamma}{C}\right) \sum_{i \neq 3} \sum c_{ij} u_i u_j \geq 0$;
so that $a_{ij}u_iu_j \geq 0$, $b_{ij}u_iu_j \geq 0$, $c_{ij}u_iu_j \geq 0$, $\gamma < \epsilon$ and $C > u_1 + u_2 + u_3$. Therefore all solutions of the system of equations (1) are contained in the region $\mathscr{D}$. □

The solution of the system (1) stabilizes in a fixed point compatible with Hardy-Weinberg equilibrium, which states that "when there is no evolutionary factor acting in a population, the frequency of alleles and genotypes remains constant". The following theorem establishes a relationship between the equilibrium point obtained from a dimensionless of the system (1) and the Hardy-Weinberg equilibrium.

Theorem 2. *The dimensionless form of system (1), without dispersal, and assuming Mendelian genetics, has an equilibrium state (5) equivalent to Hardy–Weinberg equilibrium* $(p^2, 2pq, q^2)$ *where* p *and* q *are allele frequencies of* w *and* g*, respectively.*

Proof. Let us define the new variables as $\breve{u}_i = \dfrac{u_i}{N}$, clearly $\sum \breve{u}_i = 1$. Substituting these new variables in the system (1), disregarding diffusion and taking into account that the total mosquitoes population quickly stabilizes at the equilibrium point $N^* = C\left(1 - \dfrac{\delta_2}{\gamma}\right)$, the dimensionless form of system (1) assuming coefficientes from classical genetic mendelian can be given by

$$\begin{cases} \dfrac{d\breve{u}_1}{d\tau} = \left(a_{11}\breve{u}_1 + 2a_{12}\breve{u}_2 - 1\right)\breve{u}_1 + a_{22}\breve{u}_2^2 \\ \dfrac{d\breve{u}_2}{d\tau} = \left(2b_{12}\breve{u}_1 + b_{22}\breve{u}_2 + 2b_{23}\breve{u}_3 - 1\right)\breve{u}_2 + 2b_{13}\breve{u}_1\breve{u}_3 \\ \dfrac{d\breve{u}_3}{d\tau} = c_{22}\breve{u}_2^2 + \left(2c_{23}\breve{u}_2 + c_{33}\breve{u}_3 - 1\right)\breve{u}_3 \end{cases} \tag{4}$$

where $\tau = \delta t$. Considering this dimensionless form, this model can be compared to the model obtained in Ref.[29] on the hypothesis that fitness of the three mosquitoes varieties remains the same.

To obtain the steady states of system (4), recall that $\breve{u}_1 + \breve{u}_2 + \breve{u}_3 = 1$, which means the total population remains constant. Therefore, without lost of generality, this state may be written in the following form: $(\breve{u}_1, 1 - \breve{u}_1 - \breve{u}_3, \breve{u}_3)$.

Putting $\dfrac{d\breve{u}_1}{d\tau} = \dfrac{d\breve{u}_2}{d\tau} = \dfrac{d\breve{u}_3}{d\tau} = 0$ and solving the system (4) for $\breve{u}_1$, $\breve{u}_2$, $\breve{u}_3$, taking the already known coefficients a_{ij}, b_{ij} and c_{ij}, the steady state reads:

$$(\breve{u}_1^*, \breve{u}_2^*, \breve{u}_3^*) = \left(1 + \breve{u}_3^* - 2\sqrt{\breve{u}_3^*}, -2\breve{u}_3^* + 2\sqrt{\breve{u}_3^*}, \breve{u}_3^*\right). \tag{5}$$

For each $\breve{u}_3^*$ obtained, and consequently $\breve{u}_1^*$ and $\breve{u}_2^*$, the trajectory related to the system converges to a specific equilibrium. Let us show that this result is consistent with the Hardy-Weinberg equilibrium, that states that both allele and genotype frequencies in a population remain constant, that is, they are in equilibrium from generation to generation unless specific disturbing influences are introduced, provided that the following requirements must be fulfilled: random mating, no mutation, no migration or emigration, infinitely large population size, and no selective pressure for or against any traits.

Hardy–Weinberg law establishes a relationship between genotype and allele frequencies, given by $f(w,w) = p^2$, $f(w,g) = 2pq$ and $f(g,g) = q^2$, being $p + q = 1$ and $p^2 + 2pq + q^2 = 1$, where $f(w,w)$, $f(w,g)$ and $f(g,g)$ are genotype frequencies and p and q are allele frequencies. Thus, $p = \breve{u}_1(0) + \frac{1}{2}\breve{u}_2(0)$ and $q = \breve{u}_3(0) + \frac{1}{2}\breve{u}_2(0)$.

It remains to show that the equilibrium state (5) is equivalent to Hardy–Weinberg equilibrium $(\breve{u}_1^{H-W}, \breve{u}_2^{H-W}, \breve{u}_3^{H-W}) = (p^2, 2pq, q^2)$. Putting $\breve{u}_3^* = q^2$, it implies

$$\breve{u}_3^* = \left(\breve{u}_3(0) + \breve{u}_2(0)/2\right)^2. \tag{6}$$

Introducing (6) in (5), we have

$$\breve{u}_1^* = 1 + q^2 - 2\sqrt{q^2} = 1 + (1-p)^2 - 2(1-p) = p^2. \tag{7}$$

Writing (7) in terms of initial conditions:

$$\breve{u}_1^* = \left(\breve{u}_1(0) + \breve{u}_2(0)/2\right)^2. \tag{8}$$

Analogously, for $\breve{u}_2^*$ in (5):

$$\breve{u}_2^* = -2q^2 + 2\sqrt{q^2} = 2q(1-q) = 2pq, \tag{9}$$

or equivalent:

$$\breve{u}_2^* = 2\left(\breve{u}_1(0) + \breve{u}_2(0)/2\right)\left(\breve{u}_3(0) + \breve{u}_2(0)/2\right). \tag{10}$$

□

Finally, let us investigate the behavior of solution around the equilibrium point. The next theorem shows the equilibrium of system is a stable degenerate point.

Theorem 3. *The equilibrium point (5) is stable degenerate.*

Proof. Considering $\sum \breve{u}_i = 1$, the system (4) can be reduced to

$$\frac{d\breve{u}_1}{d\tau} = \frac{d\breve{u}_3}{d\tau} = -\breve{u}_3\breve{u}_1 + \left(\frac{1 - \breve{u}_1 - \breve{u}_3}{2}\right)^2. \tag{11}$$

The linearization of system (11) around the equilibrium point (5) give the Jacobian matrix

$$J = \begin{pmatrix} -\sqrt{\breve{u}_3} & -\breve{u}_1 - \sqrt{\breve{u}_3} + \breve{u}_3 \\ -\sqrt{\breve{u}_3} & -\breve{u}_1 - \sqrt{\breve{u}_3} + \breve{u}_1 \end{pmatrix}, \tag{12}$$

whose eigenvalues are $\lambda_1 = 0$ and $\lambda_2 = \breve{u}_3 - \breve{u}_1 - 2\sqrt{\breve{u}_3}$, so that, the system has a null eigenvalue. Consequently there exists a line of equilibrium points, characterizing a degenerated case. Consequently, if the sign of λ_2 is negative, the solution tends to the line of the equilibrium point, characterizing stable equilibrium. Direction field surrounding this stable degenerate point can be seen in Fig 1. □

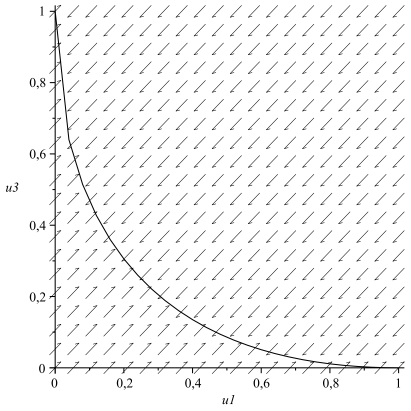

Fig 1. Direction field for Eq (11)
